# Upscaling biodiversity monitoring: Metabarcoding estimates 31,846 insect species from Malaise traps across Germany

**DOI:** 10.1111/1755-0998.14023

**Published:** 2024-10-04

**Authors:** Dominik Buchner, James S. Sinclair, Manfred Ayasse, Arne J. Beermann, Jörn Buse, Frank Dziock, Julian Enss, Mark Frenzel, Thomas Hörren, Yuanheng Li, Michael T. Monaghan, Carsten Morkel, Jörg Müller, Steffen U. Pauls, Ronny Richter, Tobias Scharnweber, Martin Sorg, Stefan Stoll, Sönke Twietmeyer, Wolfgang W. Weisser, Benedikt Wiggering, Martin Wilmking, Gerhard Zotz, Mark O. Gessner, Peter Haase, Florian Leese

**Affiliations:** ^1^ Aquatic Ecosystem Research University of Duisburg Essen Essen Germany; ^2^ Senckenberg Research Institute and Natural History Museum Frankfurt Gelnhausen Germany; ^3^ Institute of Evolutionary Ecology and Conservation Genomics University of Ulm Ulm Germany; ^4^ Centre for Water and Environmental Research (ZWU) Essen Germany; ^5^ Black Forest National Park Freudenstadt Germany; ^6^ University of Applied Sciences HTW Dresden Dresden Germany; ^7^ Entomological Society Krefeld Krefeld Germany; ^8^ Faculty of Biology University of Duisburg Essen Essen Germany; ^9^ Helmholtz Centre for Environmental Research—UFZ Department of Community Ecology Halle Germany; ^10^ Department of Evolutionary and Integrative Ecology Leibniz Institute of Freshwater Ecology and Inland Fisheries (IGB) Berlin Germany; ^11^ Institute of Biology Freie Universität Berlin Berlin Germany; ^12^ Kellerwald‐Edersee National Park Bad Wildungen Germany; ^13^ Field Station Fabrikschleichach, Department of Animal Ecology and Tropical Biology Julius‐Maximilians‐Universität Würzburg Würzburg Germany; ^14^ Bavarian Forest National Park Grafenau Germany; ^15^ Senckenberg Research Institute and Natural History Museum Frankfurt Frankfurt am Main Germany; ^16^ LOEWE Centre for Translational Biodiversity Genomics Frankfurt am Main Germany; ^17^ Institute for Insect Biotechnology Justus‐Liebig‐University Gießen Gießen Germany; ^18^ German Centre for Integrative Biodiversity Research (iDiv) Halle‐Jena‐Leipzig Leipzig Germany; ^19^ Systematic Botany and Functional Biodiversity, Institute for Biology Leipzig University Leipzig Germany; ^20^ Institute for Botany and Landscape Ecology Greifswald University Greifswald Germany; ^21^ Environmental Campus Birkenfeld, University of Applied Sciences Trier Hoppstädten‐Weiersbach Germany; ^22^ Eifel National Park Schleiden‐Gemünd Germany; ^23^ Terrestrial Ecology Research Group, Department of Life Science Systems, School of Life Sciences Technische Universität München Freising‐Weihenstephan Germany; ^24^ Lower Saxon Wadden Sea National Park Authority Wilhelmshaven Germany; ^25^ Institute of Biology and Environmental Sciences Carl von Ossietzky Universität Oldenburg Oldenburg Germany; ^26^ Department of Plankton and Microbial Ecology Leibniz Institute of Freshwater Ecology & Inland Fisheries (IGB) Stechlin Germany; ^27^ Department of Ecology Berlin Institute of Technology (TU Berlin) Berlin Germany

**Keywords:** biodiversity monitoring, DNA metabarcoding, insect diversity, Malaise trap

## Abstract

Mitigating ongoing losses of insects and their key functions (e.g. pollination) requires tracking large‐scale and long‐term community changes. However, doing so has been hindered by the high diversity of insect species that requires prohibitively high investments of time, funding and taxonomic expertise when addressed with conventional tools. Here, we show that these concerns can be addressed through a comprehensive, scalable and cost‐efficient DNA metabarcoding workflow. We use 1815 samples from 75 Malaise traps across Germany from 2019 and 2020 to demonstrate how metabarcoding can be incorporated into large‐scale insect monitoring networks for less than 50 € per sample, including supplies, labour and maintenance. We validated the detected species using two publicly available databases (GBOL and GBIF) and the judgement of taxonomic experts. With an average of 1.4 M sequence reads per sample we uncovered 10,803 validated insect species, of which 83.9% were represented by a single Operational Taxonomic Unit (OTU). We estimated another 21,043 plausible species, which we argue either lack a reference barcode or are undescribed. The total of 31,846 species is similar to the number of insect species known for Germany (~35,500). Because Malaise traps capture only a subset of insects, our approach identified many species likely unknown from Germany or new to science. Our reproducible workflow (~80% OTU‐similarity among years) provides a blueprint for large‐scale biodiversity monitoring of insects and other biodiversity components in near real time.

## 1 INTRODUCTION

Insects are the most diverse animal taxonomic group on Earth and contribute to many essential ecosystem processes and services, such as pollination, nutrient cycling and organic matter decomposition (Cardoso et al., 2020). However, insect populations are declining (Wagner, 2020) and our ability to mitigate these declines is hindered by poor understanding of spatial distributions, habitat requirements, biotic interactions, dynamics and even the overall number of extant species. About 1 million insect species have been described to date, but recent estimates of total species numbers stand at about 5.5 million (Stork, 2018) or even more (IPBES, 2019). Surprisingly, many new species are being reported even for very well‐studied and generally species‐poor areas with a long history of entomological research. For example, based on 4000 species caught with Malaise traps in Sweden, Karlsson et al. (2020) reported almost 700 insect species new to science and ~ 1300 new to Sweden. Similarly, in a DNA barcoding analysis of about 62,000 specimens collected with Malaise traps, Chimeno et al. (2022) estimated over 2000 dipteran species new to Germany, raising the total number of 33,341 known insect species in the country (Klausnitzer, 2005) to approximately 35,500. Thus, while many new insect species are being continuously reported, the vast majority still remain unknown.

A key constraint to closing the current information gap on insects is the vast number of species and specimen‐rich samples that must be processed. Terrestrial and aquatic monitoring methods, like Malaise, canopy or light traps, as well as samples collected with nets from freshwater habitats, can collect thousands of specimens (e.g. Habel et al., 2023; Karlsson et al., 2020; Resh & Jackson, 1993), many of which are small and difficult to identify even for experts, resulting in taxonomic neglect (Srivathsan et al., 2023). National and international monitoring networks, such as the Global Malaise Trap programme (Geiger et al., 2016), BioScan (Hobern, 2021), LifePlan (www.helsinki.fi/en/projects/lifeplan) or the Swedish Malaise Trap programme (Karlsson et al., 2020), have started coordinated large‐scale insect sampling initiatives based on standardized traps and procedures (e.g. Hallmann et al., 2017). However, classical taxonomic analyses of these samples cannot keep pace with the rate of collection, particularly given the low and globally declining number of taxonomic experts (European Commission, 2022). While the importance of taxonomic expertise remains undisputed, there is a clear need for alternative methods to assess insect species diversity both efficiently and reliably (Chua et al., 2023; van Klink et al., 2022).

DNA metabarcoding is one key method for assessing specimen‐rich samples. Following more than a decade of research and trial applications, metabarcoding has now reached a high technology readiness level, presenting a promising solution for examining a fuller range of insect biodiversity in large‐scale monitoring programmes. A range of suitable protocols for specimen collection, processing and data analysis are now available (Buchner, Macher, et al., 2021; Montgomery et al., 2021). This includes suitable primer pairs (Braukmann et al., 2019; Elbrecht et al., 2019) and insect barcode reference data on BOLD (Ratnasingham & Hebert, 2007). The key strength of DNA metabarcoding is that it rapidly delivers taxonomically highly resolved taxa lists, whereas obtaiquantitative information on species abundance or biomass remains a challenge (Sickel et al., 2023).

Despite its potential, implementing DNA metabarcoding for large‐scale and long‐term insect biodiversity monitoring programmes is still in its infacy. There are several reasons. In particular sample throughput is still constrained by the need for significant manual labour, expensive DNA kits and reagents, and difficulties in accessing information on lab and analysis procedures (McGee et al., 2019) and the lack of formal standards for the procedures (Chua et al., 2023). All these aspects present roadblocks to large‐scale implementation. Furthermore, incomplete and partly inconsistent reference databases impact the accuracy and quantity of species‐level assignments and thus the completeness and validity of the resulting species lists (Chua et al., 2023). This problem remains particularly pervasive for highly diverse groups like Diptera and Hymenoptera that are underrepresented in reference libraries because of those species that are difficult to distinguish based on morphological criteria. The unknown (i.e. undescribed) species in these poorly explored groups are often referred to as ‘dark taxa’ (Hartop, 2021). However, in the absence of reference barcodes for species, or even when formal species descriptions are lacking, DNA metabarcoding can still overcome these limitations using genetic distance thresholds to approximate entities that roughly reflect species, such as molecular Operational Taxonomic Units (OTUs) or Barcode Index Numbers (Ratnasingham & Hebert, 2013) (BINs). Even in the absence of species in databases, such distance‐based entities can approximate species numbers and have been applied in ecological and ecotoxicological research for many years (Beermann et al., 2018; Hoppeler et al., 2016; Sturmbauer et al., 1999).

**FIGURE 1:**
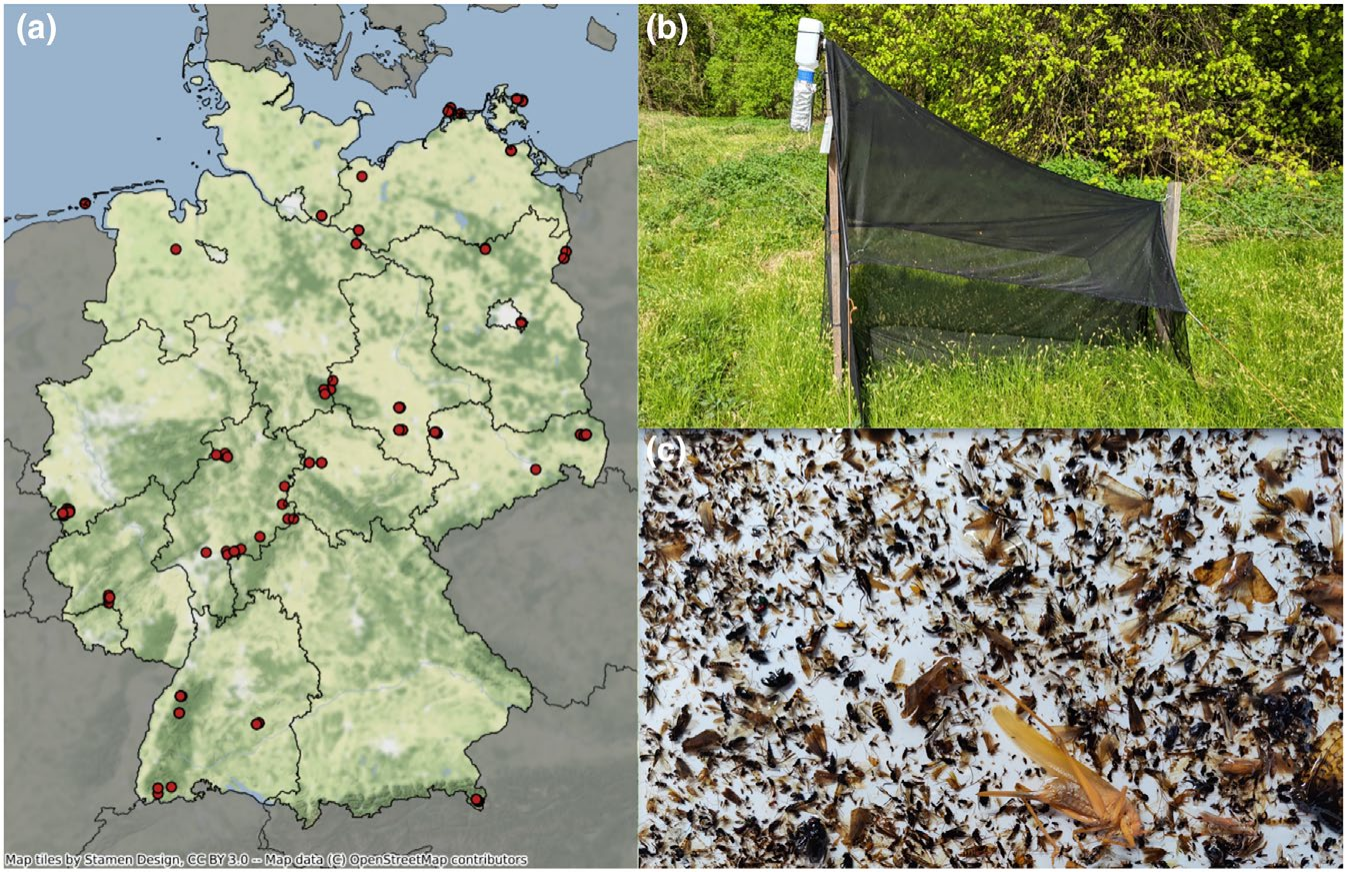
(a) Location of the in total 75 Malaise traps across Germany, (b) Lateral view of a Malaise trap with collection bottle protected from sunlight at the upper left end and (c) Top view of a preserved Malaise trap sample spread in a white tray.

DNA metabarcoding analyses to date have been limited to either a specific region within a country (e.g. Geiger et al., 2016; Habel et al., 2023; Uhler et al., 2021), short time spans (e.g. Huang et al., 2022; Li et al., 2023) or specific taxonomic groups (e.g. Huang et al., 2022). None has quantified the full extent of insect diversity—including dark taxa—at the whole‐country scale and through time. Thus, the present study fills this gap by presenting a robust DNA metabarcoding workflow for application to large‐scale insect monitoring programmes, combined with a new multi‐level procedure to assess the validity of species records. We analysed 1815 Malaise trap samples collected in 2019 and 2020 from 75 individual traps across Germany (Figure 1). Our principal aim was to (1) present the workflow as a resource for use by other researchers by providing detailed and publicly available laboratory procedures and bioinformatic programmes. Additionally, we (2) quantified the time and cost investment required per sample for this workflow to evaluate its affordability, which is a key limitation to using any metabarcoding workflow. Furthermore, we (3) evaluated the reliability of the data provided by our workflow in terms of the number of known species detected, number of dark taxa and their likely validity, and variation in species detected between years.

The results obtained with the new workflow show that DNA metabarcoding is feasible for large‐scale and long‐term insect monitoring, and providing insight into insect diversity at scales that have been challenging to study so far.

## 2 MATERIALS AND METHODS

### 2.1 Sampling

Sampling was conducted as part of the nationwide German Malaise trap monitoring programme (Welti et al., 2022) comprising forests, grassland, agricultural and urban areas (https://www.ufz.de/lter‐d/index.php?de=46285). Most of the 31 sites in which the Malaise traps were placed belong either to the German LTER‐D network (Haase et al., 2016; Mirtl et al., 2018) (Long‐Term Ecological Research) or to the network of national natural landscapes (https://nationale‐naturlandschaften.de). At each site, one to six Malaise traps have been operated to monitor biomass and species composition of flying insects in different habitats. In the present study, we used a total of 1815 Malaise trap samples from 56 locations across Germany during 2019, with 19 locations added in 2020 (Figure 1). Traps were emptied every 2 weeks from the beginning of April until the end of October in both years (approx. 15 samples per year, depending on site‐specific climatic conditions). Insects were caught in 80% denatured ethanol and their wet biomass was measured following Welti et al. (2022). Samples were kept in 96% denatured ethanol and protected from light until later genetic analysis.

### 2.2 Sample processing

All samples were divided into two size classes (small ≤4 mm; large >4 mm) to increase taxon recovery rates of small taxa (Elbrecht et al., 2021). Samples spread on a perforated plate sieve (4 mm hole diameter) were stirred using a magnetic stirrer (750 rpm) in ethanol (Figure S1), so that small individuals passed through the holes, whereas the large ones were retained on top of the sieve. The size fractions were homogenized following the protocol described in Buchner, Haase, and Leese (2021), except that the homogenization time was reduced to 30 s. The two size fractions were subsequently pooled at a ratio of 1:4 (large 200 μL:small 800 μL) as recommended by Elbrecht et al. (2021).

### 2.3 DNA extraction

Generally, the laboratory steps followed the workflow described in Buchner et al. (2021). All procedures are available as step‐by‐step protocols in a protocols.io repository (Buchner, 2022b). All subsequent steps after size‐sorting and homogenization were completed on a Biomek FX^P^ liquid handling workstation (Beckman Coulter, Brea, CA, USA). After sample lysis (Buchner, 2022e), samples were processed in duplicate during the entire library preparation to control for possible cross‐contamination. Additionally, in each 96‐well plate 12 negative controls were included. DNA was extracted using a magnetic bead protocol (Buchner, 2022a). Extraction success was verified on a 1% agarose gel. For all samples that did not amplify, the extraction was repeated with silica spin columns (Buchner, 2022a), which were always successful.

### 2.4 Sequencing library preparation

The PCR for the metabarcoding library followed a two‐step PCR protocol (Zizka et al., 2019) targeting a 205‐bp fragment (Vamos et al., 2017) of the cytochrome oxidase c subunit I (COI) gene. DNA was amplified in a first PCR using the Qiagen Multiplex Plus Kit (Qiagen, Hilden, Germany) with a final concentration of 1x Multiplex Mastermix, 200 nM of each primer (fwh2F, fwhR2n (Vamos et al., 2017)) and 1 μL of DNA, filled up with PCR‐grade water to a final volume of 10 μL. The amplification protocol was 5 min of initial denaturation at 95°C, 20 cycles of 30 s denaturation at 95°C, 90 s of annealing at 58°C and 30 s of extension at 72°C, followed by a final elongation step of 10 min at 68°C. Each of the PCR plates used in the first step was tagged with a unique combination of inline tags. Additionally, the primers contained a universal binding site for the primer used in the second PCR step to anneal (Table S1). The PCR products were purified using a bead‐based protocol and a ratio of 0.8x and an elution volume of 40 μL to remove remaining primers and potential primer dimers (Buchner, 2022c).

In the second PCR, DNA was amplified at a final concentration of 1× Multiplex Mastermix, 100 nM of each primer (Table S1), 1× Corralload Loading Dye, 2 μL of the cleaned‐up product of the first PCR in a final volume of 10 μL. The amplification protocol was 5 min of initial denaturation at 95°C, 25 cycles of 30 s denaturation at 95°C, 90 s of annealing at 61°C and 30 s of extension at 72°C, followed by a final elongation step of 10 min at 68°C. PCR success was visualized on a 1% agarose gel.

To achieve a similar sequencing depth, the PCR products were normalized to equal concentrations. Normalization was achieved with a bead‐based protocol and a ratio of 0.7× (Buchner, 2022d) and an elution volume of 40 μL. The whole volume of the normalized samples was then pooled into the final libraries. Libraries were then concentrated using a silica spin‐column protocol (Buchner, 2022a). Library concentrations were quantified on a Fragment Analyzer (High Sensitivity NGS Fragment Analysis Kit; Advanced Analytical, Ankeny, IA, USA). The libraries were sequenced at Macrogen Europe using the HiSeq X platform with a paired‐end (2 × 150 bp, 15 lanes) kit or at CeGaT (Tübingen, Germany) using the MiSeq V2 platform (2 × 150 bp, 1 lane).

### 2.5 Bioinformatics

Raw data of the sequencing runs were delivered demultiplexed by index reads. Since no differences were detected between sequencing runs, they were all pooled before subsequent analyses. Additional demultiplexing of the inline tags was achieved with the Python package ‘demultiplexer’ (v1.1.0, https://github.com/DominikBuchner/demultiplexer). Reads were further processed with the APSCALE pipeline (Buchner et al., 2022) (v1.4.0, https://github.com/DominikBuchner/apscale) using default settings. Briefly, paired‐end reads were first merged using vsearch (Rognes et al., 2016) (v2.21.1) before the primer sequences were trimmed using cutadapt (Martin, 2011) (v3.5). Only reads with a length of 205 bp (±10) and with a maximum expected error of 1 were retained. Identical reads less abundant than 4 were discarded prior to OTU clustering and globally dereplicated before OTUs were clustered based on a similarity threshold of 97%. Reads were then mapped to OTUs. This included singletons. The resulting OTU table was filtered for erroneous OTUs with the LULU algorithm (Frøslev et al., 2017) as implemented in APSCALE. Taxonomic assignment was performed using BOLDigger (Buchner & Leese, 2020) (v1.5.4, https://github.com/DominikBuchner/BOLDigger). The best hit was determined with the BOLDigger method and the API verification method. This resulted in a raw OTU table (Table S2) that was used in subsequent analysis.

### 2.6 Data filtering

To control for possible contamination during the laboratory workflow, the technical replicates of each sample, as well as the negative controls, were merged by summing up the reads, provided that the reads were present in both replicates. Subsequently, the maximum number of reads per OTU present in all of the negative controls was subtracted from the respective OTU (Table S3). All OTUs were analysed for stop‐codons, and any OTU containing stop‐codons were removed. We analysed two datasets: (1) OTUs assigned at the species level and (2) OTUs assigned to insects. To further clean dataset 1, all OTUs sharing species assignments were merged by summing up their reads. Retrieved species names containing numbers or punctuation marks were also removed (e.g. incomplete database records). The resulting final species list (Table S4) for all samples was used for the validation procedure.

### 2.7 Validation of taxonomic assignment

To validate the resulting species‐level list, three different approaches were used. First, the occurrences in conjunction with their specific locations of all named species were checked for plausibility by taxonomic experts at the Entomological Society Krefeld, Germany. As a basis, the experts used the digitally available insect species catalogue from the Entomofauna Germanica (Klausnitzer, 2005), which is best resembled through the list from the German Barcode of Life portal (https://gbol.bolgermany.de). This plausibility check included fixing problems related to synonymy and incorporated the primary scientific literature available to the experts. Second, all detected named species were checked for GBIF records within a 200‐km radius around the given trap. This value was selected to be sufficiently high to accommodate the often patchy and rare species records in GBIF, the large size of the study area and the fact that many flying insect species are highly mobile. To do so, a polygon was drawn around all trap locations with occurrences of the respective species, and the border of this polygon was then extended by 200 km (Figure S2). Records were extracted from the GBIF database using the ‘rgbif’ package (Chamberlain et al., 2022). Lastly, all named species were checked against the German Barcode of Life database, which was downloaded with a custom Python script (Script S1). If a species name occurred in the German Barcode of Life database, it was accepted as valid. Additionally, this step potentially identified species that are unlikely to occur in Germany, supplementing the information obtained from GBIF. A record was accepted as valid when two of the three validation criteria were met, which also helps to control for false positives (Table S5). Such multi‐critera validation approaches have been used elsewhere (Pereira et al., 2021).

### 2.8 Statistical analysis

To assess if sequencing depth was sufficient across all samples, we conducted a read‐based rarefaction using a custom python script. This approach involved randomly sampling reads without replacement from each sample in increments of 0.1% (with 50 iterations per step) of the total read count. Subsequently, we fitted a Michaelis–Menten‐type equation to the resulting rarefaction curve. Using this function, we computed OTU richness when doubling the sequencing depth. If OTU richness increased by less than 5% when doubling the sequencing depth, the sequencing depth was considered sufficient (Figure S3; Table S6).

In addition to examining sequencing rarefaction curves, we also examined rarefaction curves for both validated and plausible species using the _i_Chao2 (Chiu et al., 2014) estimator. We did so to determine the potential influence of collecting more samples from each site, such as by sampling for a longer time period or more frequently, on the number of detected insect species. This was done by randomly drawing subsets of samples (50 iterations each) from the dataset without replacement in increments of 5 from 5 to 1815.

### 2.9 Plausible species and dark taxa estimation

To address the potential over‐estimation of species diversity based on OTU numbers we computed the mean number of OTUs per validated species for each of the 20 insect orders within the dataset. This mean was then used to normalize the OTU count for each order. Specifically, we divided the number of OTUs per order by the calculated mean, providing an estimate of additional species present in the dataset that have not yet been assigned a species name. We refer to these additional species as plausible species. Furthermore, we obtained data from the Barcode of Life data systems (accession date: 14 April 2023), including the number of species barcoded with a voucher specimen collected in Germany, along with the total number insect species known from Germany (Klausnitzer, 2005). This additional information allowed us to distinguish plausible species lacking a reference sequence from potential dark data within the dataset. The described correction was performed on order as well as on family level, but for simplicity we focused the subsequent analysis on the order level.

### 2.10 Time and cost estimations

To estimate the time and costs needed for our nationwide insect diversity assessment via metabarcoding, we used vendor list prices for materials, as well as runtimes on the liquid handler plus estimates for laboratory set‐up before and after each step of the workflow. These costs include all labware and chemicals needed to complete the respective step of the protocol. Incubation times are not included in the time estimates because the time can be used to process other samples. The calculated price per sample is based on 1815 samples including replicates and negative controls, resulting in a total number of 4149 individual reactions spread across 44 96‐well PCR plates. A total of 10 blenders were used for homogenization, which are included in the cost estimate. For sequencing, cost estimates are based on the current costs of 1100 € for 110 Gb output at a commercial service provider (Macrogen Europe) using the NovaSeq 6000 S4, which has replaced the HiSeq platform, and a sequencing depth of at least 1.5 million reads per sample (~817 Gb). Labour costs were estimated at 60 € per hour, corresponding to an experienced scientist in Germany. The costs for the use of the liquid handler were estimated based on a linear depreciation over a total expected lifetime of 10 years. Annual gross maintenance costs of 40,000 € were assumed for instruments, resulting in total depreciation and maintenance costs of approximately 4000 € for the present study. No rental and auxiliary costs needed to be applied in this study.

## 3 RESULTS

### 3.1 Sequencing results and species validation

We performed high‐throughput DNA metabarcoding including replicates and negative controls on 1815 Malaise trap insect samples (775 for 2019 and 1040 for 2020; Table 1) using a 205‐bp fragment of the mitochondrial cytochrome *c* oxidase I (COI) gene as a marker. All samples and sequencing runs combined yielded 3,999,082,169 demultiplexed read pairs. The average read number per sample in the final read table was 1,401,469 (±SD of 618,631). Sequencing depth was sufficient for all samples, with a mean increase in richness of 0.17% by doubling the sequencing depth (0%–1.46%; Table S6).

**TABLE 1:** Summary statistics of OTUs, assigned insect species and OTUs assigned to validated insect species according to the two‐out‐of‐three validation criterion.

	2019 (*n* = 775)	2020 (*n* = 1040)	Σ (*n* = 1815)
Raw OTUs	41,189	50,166	52,981
Insect OTUs	39,367	47,652	50,087
Insect species	9728	11,183	11,776
Validated species according to:			
Expert validation	9132	10,475	11,030
GBIF validation	7969	8867	9254
GBOL validation	8777	10,056	10,574
Validated species	8983	10,276	10,803
Plausible species			21,043
Total species			31,846

*Note*: Plausible species calculated from the average number of OTUs per validated species and family. *n* = number of Malaise trap samples.

Abbreviation: OTUs, Operational Taxonomic Units.

Sequencing yielded a total of 52,981 raw OTUs, 50,087 of which were assigned to insects. We were able to assign 11,776 species names to a total of 15,042 OTUs. Two thirds of these species (10,803) were validated via three different criteria involving (i) expert judgement from entomologists with particular knowledge of long‐term Malaise trap community data from German Malaise traps, (ii) a comparison with an online database that includes the known German species (Klausnitzer, 2005) (GBOL) and (iii) a GBIF record check within a 200‐km radius. Species were regarded as ‘validated’ when two of the three validation criteria supported them. The three different validation criteria showed a pairwise agreement exceeding 80%. Taxonomic experts validated an additional 355 species not yet not in the list of species reported from Germany (Table 1), leading to an overlap of 97% with the GBOL database. GBIF and taxonomic expert validation had an overlap of 84%, and GBIF and GBOL of 82% (Table S7). Consequently, a total of 35,045 insect OTUs remained unassigned, either because species‐level reference data are lacking or the species are truly unknown (i.e. dark taxa; Table 1), resulting in a large discrepancy between the number of named species and recorded insect OTUs. Although sometimes several OTUs were assigned to the same species, 9061 (83.9%) of our validated species were represented by a single OTU (Table S8).

### 3.2 Species richness estimation

**FIGURE 2:**
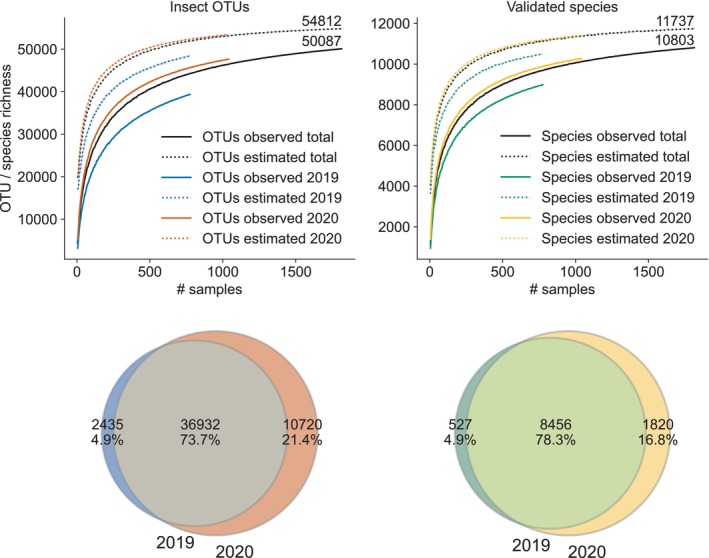
Top row: Rarefaction curves showing richness of insect OTUs and validated species richness that were either observed (solid lines) or estimated (dotted lines). Bottom row: Shared and unique absolute numbers and proportions of the insect OTUs (left circles) and validated insect species (right circles) collected in 2019 and 2020.

Our sampling effort captured the majority of OTUs. Based on rarefaction curves, a greatly increased sampling effort in each site, for instance by sampling more frequently or for a longer period at all sites, may have only resulted in detecting an additional 4725 OTUs (+9.4%) and 934 validated species (+8.6%; Figure 2). Additionally, most of the OTUs were found in both sampling years (36,932 = 73.7%) with more insect OTUs occurring exclusively in 2020 (10,720) than in 2019 (2435). The same is true for the validated insect species. Most were found in both years (8456 = 78.3%) but more than three times as many occurred exclusively in 2020 (1820) compared to 2019 (527).

### 3.3 OTU distribution across insect groups and unknown taxa

**FIGURE 3:**
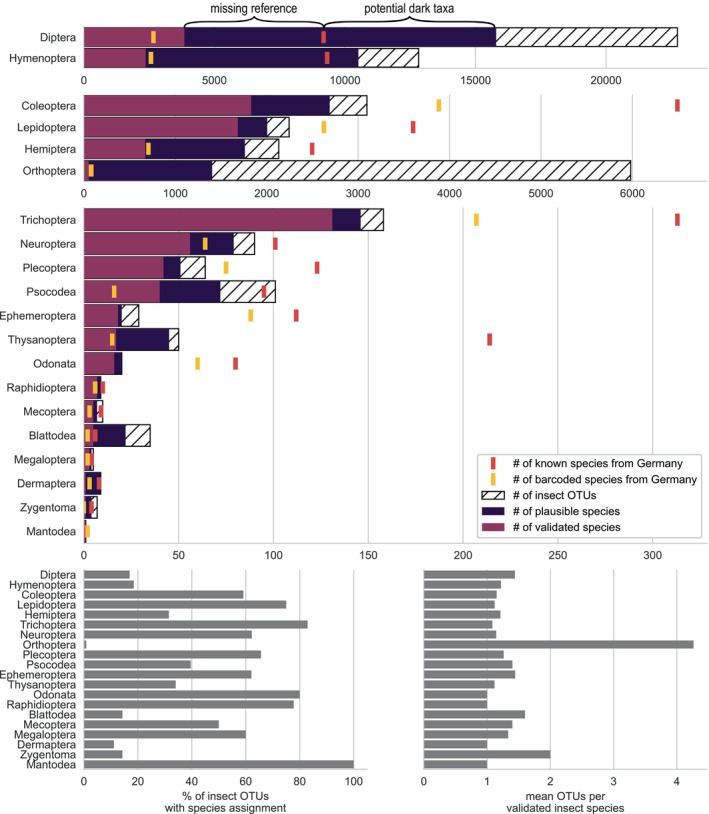
Top panels: Number of total insect OTUs, plausible and validated insect species per order identified in the present study compared to the total number of species reported from Germany or sampled in Germany and included in the BOLD database (data accessed on 14 April 2023). Bottom left panel: Percentage of OTUs assigned to species. Bottom right panel: Mean number of distinct OTUs assigned to a given plausible species.

The four most diverse insect orders in Germany (Diptera, Hymenoptera, Lepidoptera and Coleoptera) were well represented in the dataset (Figure 3, top row). Diptera and Hymenoptera were the most common orders both at the OTU (22,732 = 45.4% and 12,823 = 25.6%) and species level (3851 = 35.6% and 2370 = 21.9%). The 10,803 validated insect species represent 33.5% of the 35,500 insect species known in Germany (Klausnitzer, 2005) and 82.6% of the 13,076 barcoded insect species recorded in the country (Table S9).

The percentage of OTUs assigned to named species differed considerably among insect orders, being highest for Lepidoptera (75%), followed by Coleoptera (59%). Less than a fifth of the OTUs identified as Diptera and Hymenoptera could be assigned names at the species‐level (17% and 18% respectively). The lowest percentage of OTUs assigned to named species was for Orthoptera (<1%) (Figure 3, bottom left panel). The mean number of OTUs per validated insect species varied slightly among insect orders and families, typically between 1 and 1.5, but Orthoptera (specifically the Acrididae) were represented by >4 and Zygentoma (specifically Lepismatidae) by ~2 OTUs per species (Figure 3, bottom right panel).

Based on the mean number of OTUs per validated insect species calculated at either the order or family level, we estimated that our data set respectively included either an additional 22,496 or 21,043 plausible insect species (Table S10). All OTUs belonging to Orthoptera were removed for these estimates to avoid artificially inflating the total number of species (Information S1). The lower of the two numbers is a more conservative estimate, but since we identified 435 different families (see Table S11 for further family level information), our analysis focuses, for simplicity reasons, on the order level. The additional plausible species could be those that either (a) could not be identified to species level due to a lack of a reference in the present reference database, (b) had not previously been recorded from Germany or (c) represent new species to science. Examples of species not reported from Germany or new to science (‘potential dark taxa’ in Figure 3; Table S10) were particularly evident in the Diptera and Hymenoptera, for which we respectively found ~6600 and ~1200 species, while the missing reference data aspect affected the assignment in all orders except the Mantodea (with 1 species only).

### 3.4 Time and cost estimation

The total costs of the laboratory workflow for duplicate sample processing and negative controls—including all needed labware, chemicals, sequencing and salaries—were estimated at 88,000 €, equivalent to about 46 € per sample (Table 2; Table S12). Costs for laboratory materials accounted for 12 € (26%) of the total costs per sample, and salaries for 34 € (74%). Sequencing was the most expensive step (contributing 4.85 € per sample), followed by enzymatic steps such as PCR (1.65 €) and sample lysis (1.54 €). The total processing time for all 1815 samples was 1030 working hours or 27.5 weeks, equivalent to 141 samples within 2 weeks for one person working full time and supported by one liquid handling robot. Most of the processing time was needed for sample size‐sorting and homogenization. All subsequent steps were completed within 6 weeks.

**TABLE 2:** Cost estimates for the proposed workflow: Cost estimates are based on vendor list prices in 2022 where available.

Processing step	Material costs per sample [€]	Total material costs [€]	Labour costs per sample [€]	Time per sample [h:min:s]	Total time [h:min:s]	Total costs [€]
Size‐sorting	—	—	15.09	0:15:00	453:45:00	27,383.81
Homogenization	0.21	379.90	11.50	0:11:26	345:45:00	21,252.45
Sample lysis	1.54	2795.64	1.91	0:01:54	57:37:30	6264.26
DNA extraction	1.02	1845.55	1.09	0:01:05	33:00:00	3823.27
QA DNA extraction	0.04	78.48	0.74	0:00:44	22:00:00	1417.24
1st PCR	1.65	2994.43	0.25	0:00:15	07:20:00	3450.83
PCR clean‐up	0.50	898.46	0.74	0:00:44	22:00:00	2237.22
2nd PCR	1.65	2994.43	0.37	0:00:22	11:00:00	3663.81
Normalization	0.47	844.47	0.74	0:00:44	22:00:00	2183.23
Pooling	<0.01	0.45	0.13	0:00:08	04:00:00	243.86
QC libraries	<0.01	10.86	0.13	0:00:08	04:00:00	254.27
Sequencing	4.85	8800.00	—	—	—	8800.00
Bioinformatic analysis	—	—	1.59	0:01:35	48:00:00	2890.51
Liquid handler depreciation and maintenance			—	—	—	4000.00
Total	11.92	21,642.67	34.28	34:04	1030:27:00	87,864.76

*Note*: Time estimates are either hands‐on time in the laboratory or mean runtimes of the robotic protocols. Costs and time required per sample are based on 1815 samples. Total time and total costs are based on the respective number of replicates processed in each step. The bioinformatic analysis was performed on a 128 CPU server with 430 Gb of memory.

## 4 DISCUSSION

### 4.1 A metabarcoding workflow for large‐scale biodiversity monitoring

We here present a scalable and cost‐efficient DNA metabarcoding workflow which allows analysing thousands of specimen‐ and species‐rich samples within several weeks to months depending on the available workforce. The proposed DNA metabarcoding workflow differs from others (e.g. Braukmann et al., 2019; Hardulak et al., 2020; Hausmann et al., 2020) in several important aspects that are key to high sample throughput at reduced costs and processing time while ensuring high‐quality data. Key differences include: (i) homogenization of samples in preservative liquid to avoid a time‐consuming drying step (Buchner, Haase, & Leese, 2021); (ii) processing of all samples in duplicate before DNA extraction as an essential quality assurance measure to reduce the probability of false positive signals; (iii) completion of all laboratory procedures by an automated liquid handling robot to minimize processing time (Buchner, Macher, et al., 2021) (except for the gel electrophoresis) and maximize consistency; (iv) a mean sequencing depth increased to ~1.4 M reads per sample to boost species detectability and (v) publication of all laboratory procedures as open‐source protocols (Buchner, 2022b) or programmes (Buchner et al., 2022; Buchner & Leese, 2020) to ensure full transparency and reproducibility following the FAIR principles (Wilkinson et al., 2016).

Labour and material costs of metabarcoding protocols are often considered prohibitively high for large‐scale monitoring programmes (Borrell et al., 2017; Montgomery et al., 2021), frequently exceeding 200 € per sample and up to 400 € (Aylagas et al., 2018; Elbrecht et al., 2017; Ji et al., 2013). The workflow presented here considerably reduces these costs, down to <50 €. This is primarily achieved by automating crucial laboratory steps and by preparing all required solutions instead of purchasing expensive commercial kits. Costs could be further cut in future large‐scale programmes by ordering chemicals and consumables in bulk and by further reducing reaction volumes for all laboratory steps involving enzymes, wherever possible (Buchner, Beermann, et al., 2021). An important consideration to lower labour costs is to reduce the processing time per sample, notably for size‐sorting and homogenization, ideally by automating these steps as well. Furthermore, expenses related to sampling in the field are not included in our analysis, although they can easily match laboratory costs. For example, in our monitoring programme, costs for Malaise trap installation, maintenance and biweekly sampling from April to October (15 samples per site and year in total) amounted to about 12 working hours or 240 € for a student aid. Adding travel time (highly variable but assumed here to average 15 h or 300 €) and material (approx. 450 €) would result in total costs for field sampling of 66 € per sample, unless much of the work is accomplished by volunteers. This sum ignores expenses for rent and additional costs (heating, electricity, etc.), which need to be added when commercial providers are solicited.

Another crucial but often neglected step in metabarcoding workflows is species validation. Validating species records poses a significant challenge for many taxa due to the dearth of reference sequences, few checklists and experts, and diverse algorithmic approaches (Hleap et al., 2021). However, these challenges could be partly addressed by using publicly available species databases, as suggested by the remarkably close agreement we found among our three validation procedures of insect experts, GBIF and GBOL matching. The taxonomic experts added further species to the list of known species from Germany based on recent scientific evidence. This agreement suggests that relying solely on public databases, like GBIF (Telenius, 2011) and the GBOL database, which most closely resembles the reports of the Entomofauna Germanica (Klausnitzer, 2005), is sufficiently accurate for large‐scale monitoring where expert validation tends to be prohibitive. This is not to advocate disregarding expert knowledge, which is critical to minimize the well‐known errors inherent in automated validation methods (e.g. Meiklejohn et al., 2019). Instead, we would like to raise awareness that acceptable alternatives exist to overcome time constraints, as they typically arise in large biodiversity monitoring programmes. FAIR and curated reference databases with up‐to‐date taxonomic names and integration of synonyms is essential for harmonized biodiversity monitoring and establishing such systems is a key challenge (Keck & Altermatt, 2023).

### 4.2 A reliable account of biodiversity

Rarefaction indicates that the 10,803 named insect species identified with our protocol would increase by only 934 species (8.6%) if sampling efforts were ramped up at the established study sites. However, we acknowledge that higher species numbers would be obtained if more sites were sampled, in particular including new habitat types. This expectation is consistent with the larger number of validated insect species in 2020 (+14.4%) when 19 more sites were sampled compared to 2019. The 10,803 detected named species represent one‐third of all insect species reported from Germany and ~83.1% of all barcoded insects from this country. This includes nearly 100% of the up‐to‐date barcoded Hymenoptera and Hemiptera, indicating that with just 75 sampling sites our approach captures a large portion of the known insect biodiversity of Germany. Other metabarcoding studies from regions within Germany have identified ~5900 species (Uhler et al., 2021) and ~11,984 insect OTUs (Habel et al., 2023). Similarly, a DNA barcoding study investigating 1% of the samples from the Swedish Malaise Trap programme found >4000 species (Karlsson et al., 2020), and another recent DNA barcoding study based on Malaise traps across eight countries from four continents found >25,000 species (Srivathsan et al., 2023). While we acknowledge that comparisons of insect species and OTUs numbers among studies are difficult due to differences in protocols, these examples indicate, along with the high proportion of known or barcoded insects from Germany, that rapid and cost effective metabarcoding can provide a robust inventory of biodiversity.

At the scale of our nationwide sampling network, we consistently found the same species in two consecutive years (~75% species and ~80% OTU‐similarity among years), highlighting the spatiotemporal reliability of taxa lists derived from metabarcoding when used in large‐scale monitoring. One of the principal concerns in large‐scale sampling is that high community variability in space and time, and sampling idiosyncrasies, make it difficult to detect the same species even in spatially or temporally proximate samples. This issue is particularly relevant when using methods, like metabarcoding, that can identify thousands of species, most of which will be rare and so may only occur sporadically in a single site (Jeliazkov et al., 2022). For example, traps just tens of metres apart (e.g. Steinke et al., 2021) or in successive sampling periods (e.g. Sinclair et al., in prep, Information S1) can capture very different insect communities. However, the between‐year consistency of our data suggests this issue may diminish as the spatial scale of sampling for metabarcoding increases, such as in larger‐scale biodiversity monitoring conducted across whole countries.

### 4.3 Assessing dark taxa biodiversity

A key finding of our study is the discovery of approximately five times more insect OTUs than validated insect species, demonstrating the potential value of cost‐effective metabarcoding for uncovering as of yet unknown biodiversity in large‐scale monitoring. One explanation for the higher number of OTUs we found is that many unassigned OTUs represent described species that cannot be assigned to the species level because reference sequences are lacking. Alternatively, many of these OTUs could represent dark taxa, that is, species new to science, as highlighted by Karlsson et al. (2020) and Srivathsan et al. (2023). While it is difficult to estimate the number of OTUs attributed to either known species without a reference barcode or to new species, we can roughly quantify this by comparing OTU‐to‐species ratios. Our mean number of OTUs per validated insect species of 1.0–1.5 suggests that our chosen cut‐off of 3% sequence identity generally delineates different species accurately. The only exception is the Orthoptera, which are known to exhibit many pseudogenes (see Information S1) and that would inflate the OTU‐to‐species ratio. Specific correction factors for different taxonomic groups can also be estimated to infer the portion of undescribed species in the OTU datasets, which our results suggest is a large proportion. This is particularly true for the Hymenoptera and Diptera, where, after correcting the OTU numbers, we estimate about 6600 and 1200 potential new species to science for the two groups. This result agrees with reports from the Swedish Malaise Trap programme where most of the observed 700 species new to science (Karlsson et al., 2020) belonged to Diptera and Hymenoptera (Ronquist et al., 2020). However, our estimate of putatively undiscovered species in Germany is obviously a significant underrepresentation for two main reasons: First, while Malaise traps capture many different insect species, they likely miss many non‐flying insects, insects flying high above ground, for example, in tree canopies, or avoiding or escaping Malaise traps (e.g. Habel et al., 2023). Second, while our 75 Malaise traps reflect almost entirely the north–south and east–west gradients of Germany, they do not cover all regions in the country. Consequently, there is likely a trove of undiscovered species, even in countries like Germany and Sweden with a relatively species‐poor fauna and a long taxonomic tradition. DNA metabarcoding can help discover the extent of this unknown biodiversity and identify taxa and sites that require additional taxonomic work.

### 4.4 Technical challenges and ways forward

Metabarcoding comes with some drawbacks and technical challenges that must be considered when proposing future insect biodiversity monitoring strategies (Chua et al., 2023). First, our approach is a compromise between improving feasibility for large‐scale monitoring and the chances of detecting all taxa. Additional sample processing, such as complementary mild lysis (Iwaszkiewicz‐Eggebrecht et al., 2023; Marquina et al., 2022), greater replication (Zizka, Geiger, et al., 2022) and using multiple gene markers or primers (Elbrecht et al., 2019; Hajibabaei et al., 2019), as well as increasing sequencing depth (this study), would undoubtedly recover more species. However, the additional effort required must be weighed against the extra information gained, which may not be substantial (Buchner, Haase, & Leese, 2021). Second, the incompleteness of regional and interregional reference sequence databases, and the generation of reference sequences of unknown species, remains a persistent challenge (e.g. Chua et al., 2023; Karlsson et al., 2020). Poorly studied groups present the largest hurdle, such as Diptera, Hymenoptera and various beetle families. Thus, to implement and facilitate insect biodiversity monitoring at large scales a clear roadmap is needed. First, DNA metabarcoding could be used to monitor insect biodiversity at hundreds of sites to identify the priority sites. Second, in‐depth analysis are needed at priority sites utilizing high‐throughput single‐specimen barcoding (Hebert et al., 2018; Meier et al., 2016) coupled with additional methods to gain insights into abundance, biomass and phenotypic data (Høye et al., 2021; Wührl et al., 2022). Improved collaboration between taxonomists, molecular ecologists but also experts from other fields, such as computer vision and deep learning, are needed to implement this roadmap of large‐scale biodiversity monitoring. Specifically, sequence data need to be linked with valid taxonomic names and undescribed species need to be described (Hartop et al., 2022). An additional advantage of incorporating these methods is their ability to provide data on species abundance and biomass, thereby complementing the biodiversity data for priority sites identified through DNA metabarcoding. Alternatively, techniques like mild lysis need to be further developed, to allow a similar high coverage of species directly from the metabarcoding bulk samples while also allowing for subsequent morphological identification. Vouchers would also help obtain the ecological trait information needed to link biodiversity changes with ecosystem function changes. Here, digital specimen vouchers have the benefit of massively increasing analysis speed. Lastly, a key advantage of molecular methods is that tissue and DNA samples can easily be stored in miniaturized formats and reanalysed in the future (Zizka, Koschorreck, et al., 2022). Emerging methods such as metagenomics can thus be applied in the future to stored samples to gain additional insights through reanalysis by eliminating biases introduced by PCR. This also makes direct intercalibration of different methods possible enabling harmonized biodiversity monitoring despite methodological advancements.

### 4.5 Implications beyond insect monitoring

We used insects collected with Malaise traps to demonstrate the value of a new DNA metabarcoding workflow that is reliable, scalable, fast, cost‐effective and particularly well‐suited for large‐scale monitoring of highly diverse taxonomic groups. The approach is, however, by no means limited to insects from Malaise traps, given that many of the key advances we highlight (e.g. automated workflow, robust species validation) are applicable to a variety of sampling methods, other invertebrate and vertebrate taxa, and even environmental DNA from various sources including soil and sediment (Pawlowski et al., 2022), by aligning all post‐sampling processing steps with the requirements for robotic high sample throughput (Buchner, Macher, et al., 2021). The presented workflow thus moves us closer to realizing the overall vision for metabarcoding, that is, to generate and link high‐throughput biodiversity analyses with large‐scale monitoring (Bush et al., 2017). Such integration would greatly enhance assessments of the massive ongoing changes in global biodiversity experienced at the present (e.g. Sinclair et al., in prep, Information S2) and biodiversity protection (e.g. the Kunming‐Montreal Global Biodiversity Framework of the CBD), including Red List and invasive species assessments as part of policy frameworks on biodiversity conservation (e.g. Wetzel et al., 2015). As demonstrated here, integrating metabarcoding into large‐scale monitoring networks is a powerful means to improving our understanding of biodiversity change and supporting conservation actions.

## AUTHOR CONTRIBUTIONS

DB, FL and PH conceived the study. DB, JSS and AJB designed and performed the analyses. DBwrote the manuscript with contributions from all authors. DB performed the laboratory processing and the sequence analysis with help from FL and YL. All authors revised and approved the manuscript. TH and MS checked the taxalist for plausibility. MOG, JM, SUP, SS, JB, JE, TH, CM, RR, TS, ST, WWW, BW, MW, FD, MF, MTM and GZ set up Malaise traps in their respective regions, maintained traps and collected samples and contributed to design and interpretation of results.

## CONFLICT OF INTEREST STATEMENT

The authors declare no conflicts of interest.

## Supporting information


Appendix S1.

## Data Availability

Demultiplexed raw read data for this study have been uploaded to the European Nucleotide Archive under the accession number PRJEB71324.

## References

[men14023-bib-0001] Aylagas, E. , Borja, Á. , Muxika, I. , & Rodríguez‐Ezpeleta, N. (2018). Adapting metabarcoding‐based benthic biomonitoring into routine marine ecological status assessment networks. Ecological Indicators, 95, 194–202. 10.1016/j.ecolind.2018.07.044

[men14023-bib-0002] Beermann, A. J. , Zizka, V. M. A. , Elbrecht, V. , Baranov, V. , & Leese, F. (2018). DNA metabarcoding reveals the complex and hidden responses of chironomids to multiple stressors. Environmental Sciences Europe, 30, 26. 10.1186/s12302-018-0157-x

[men14023-bib-0003] Borrell, Y. J. , Miralles, L. , Huu, H. D. , Mohammed‐Geba, K. , & Garcia‐Vazquez, E. (2017). DNA in a bottle—Rapid metabarcoding survey for early alerts of invasive species in ports. PLoS One, 12, e0183347. 10.1371/journal.pone.0183347 28873426 PMC5584753

[men14023-bib-0004] Braukmann, T. W. A. , Ivanova, N. V. , Prosser, S. W. J. , Elbrecht, V. , Steinke, D. , Ratnasingham, S. , de Waard, J. R. , Sones, J. E. , Zakharov, E. V. , & Hebert, P. D. N. (2019). Metabarcoding a diverse arthropod mock community. Molecular Ecology Resources, 19, 711–727. 10.1111/1755-0998.13008 30779309 PMC6850013

[men14023-bib-0005] Buchner, D. (2022a). Guanidine‐based DNA extraction with silica‐coated beads or silica spin columns . 10.17504/protocols.io.eq2ly73mmlx9/v2

[men14023-bib-0006] Buchner, D. (2022b). Invertebrate bulk sample metabarcoding protocol collection . 10.17504/protocols.io.j8nlkw4n6l5r/v4

[men14023-bib-0007] Buchner, D. (2022c). PCR cleanup and size selection with magnetic beads . 10.17504/protocols.io.36wgqj45xvk5/v3

[men14023-bib-0008] Buchner, D. (2022d). PCR normalization and size selection with magnetic beads . 10.17504/protocols.io.q26g7y859gwz/v3

[men14023-bib-0009] Buchner, D. (2022e). Sample preparation and lysis of homogenized malaise trap samples . 10.17504/protocols.io.dm6gpjrmjgzp/v1

[men14023-bib-0012] Buchner, D. , & Leese, F. (2020). BOLDigger—A python package to identify and organise sequences with the barcode of life data systems. Metabarcoding and Metagenomics, 4, e53535. 10.3897/mbmg.4.53535

[men14023-bib-0010] Buchner, D. , Beermann, A. J. , Leese, F. , & Weiss, M. (2021). Cooking small and large portions of “biodiversity‐soup”: Miniaturized DNA metabarcoding PCRs perform as good as large‐volume PCRs. Ecology and Evolution, 11, 9092–9099. 10.1002/ece3.7753

[men14023-bib-0011] Buchner, D. , Haase, P. , & Leese, F. (2021). Wet grinding of invertebrate bulk samples—A scalable and cost‐efficient protocol for metabarcoding and metagenomics. Metabarcoding and Metagenomics, 5, e67533. 10.3897/mbmg.5.67533

[men14023-bib-0014] Buchner, D. , Macher, T.‐H. , & Leese, F. (2022). APSCALE: Advanced pipeline for simple yet comprehensive analyses of DNA metabarcoding data. Bioinformatics, 38, 4817–4819. 10.1093/bioinformatics/btac588 36029248 PMC9563694

[men14023-bib-0013] Buchner, D. , Macher, T.‐H. , Beermann, A. J. , Werner, M.‐T. , & Leese, F. (2021). Standardized high‐throughput biomonitoring using DNA metabarcoding: Strategies for the adoption of automated liquid handlers. Environmental Science and Ecotechnology, 8, 100122. 10.1016/j.ese.2021.100122 36156998 PMC9488008

[men14023-bib-0015] Bush, A. , Sollmann, R. , Wilting, A. , Bohmann, K. , Cole, B. , Balzter, H. , Martius, C. , Zlinszky, A. , Calvignac‐Spencer, S. , Cobbold, C. A. , Dawson, T. P. , Emerson, B. C. , Ferrier, S. , Gilbert, M. T. P. , Herold, M. , Jones, L. , Leendertz, F. H. , Matthews, L. , Millington, J. D. A. , … Yu, D. W. (2017). Connecting Earth observation to high‐throughput biodiversity data. Nature Ecology & Evolution, 1, 1–9. 10.1038/s41559-017-0176 28812589

[men14023-bib-0016] Cardoso, P. , Barton, P. S. , Birkhofer, K. , Chichorro, F. , Deacon, C. , Fartmann, T. , Fukushima, C. S. , Gaigher, R. , Habel, J. C. , Hallmann, C. A. , Hill, M. J. , Hochkirch, A. , Kwak, M. L. , Mammola, S. , Ari Noriega, J. , Orfinger, A. B. , Pedraza, F. , Pryke, J. S. , Roque, F. O. , … Samways, M. J. (2020). Scientists' warning to humanity on insect extinctions. Biological Conservation, 242, 108426. 10.1016/j.biocon.2020.108426

[men14023-bib-0017] Chamberlain, S. , Oldoni, D. , & Waller, J. (2022). rgbif: Interface to the global biodiversity information facility API . 10.5281/zenodo.6023735

[men14023-bib-0018] Chimeno, C. , Hausmann, A. , Schmidt, S. , Raupach, M. J. , Doczkal, D. , Baranov, V. , Hübner, J. , Höcherl, A. , Albrecht, R. , Jaschhof, M. , Haszprunar, G. , & Hebert, P. D. N. (2022). Peering into the darkness: DNA barcoding reveals surprisingly high diversity of unknown species of Diptera (Insecta) in Germany. Insects, 13, 82. 10.3390/insects13010082 35055925 PMC8779287

[men14023-bib-0019] Chiu, C.‐H. , Wang, Y.‐T. , Walther, B. A. , & Chao, A. (2014). An improved nonparametric lower bound of species richness via a modified good–turing frequency formula. Biometrics, 70, 671–682. 10.1111/biom.12200 24945937

[men14023-bib-0020] Chua, P. Y. S. , Bourlat, S. J. , Ferguson, C. , Korlevic, P. , Zhao, L. , Ekrem, T. , Meier, R. , & Lawniczak, M. K. N. (2023). Future of DNA‐based insect monitoring. Trends in Genetics, 39, 531–544. 10.1016/j.tig.2023.02.012 36907721

[men14023-bib-0021] Directorate‐General for Environment (European Commission) , Hochkirch, A. , Casino, A. , Penev, L. , Allen, D. , Tilley, L. , Georgiev, T. , Gospodinov, K. , & Barov, B. (2022). European Red List of insect taxonomists. Publications Office of the European Union, LU. 10.2779/364246

[men14023-bib-0022] Elbrecht, V. , Bourlat, S. J. , Hörren, T. , Lindner, A. , Mordente, A. , Noll, N. W. , Schäffler, L. , Sorg, M. , & Zizka, V. M. A. (2021). Pooling size sorted Malaise trap fractions to maximize taxon recovery with metabarcoding. PeerJ, 9, e12177. 10.7717/peerj.12177 34707928 PMC8500090

[men14023-bib-0023] Elbrecht, V. , Braukmann, T. W. A. , Ivanova, N. V. , Prosser, S. W. J. , Hajibabaei, M. , Wright, M. , Zakharov, E. V. , Hebert, P. D. N. , & Steinke, D. (2019). Validation of COI metabarcoding primers for terrestrial arthropods. PeerJ, 7, e7745. 10.7717/peerj.7745 31608170 PMC6786254

[men14023-bib-0024] Elbrecht, V. , Vamos, E. E. , Meissner, K. , Aroviita, J. , & Leese, F. (2017). Assessing strengths and weaknesses of DNA metabarcoding‐based macroinvertebrate identification for routine stream monitoring. Methods in Ecology and Evolution, 8, 1265–1275. 10.1111/2041-210X.12789

[men14023-bib-0025] Frøslev, T. G. , Kjøller, R. , Bruun, H. H. , Ejrnæs, R. , Brunbjerg, A. K. , Pietroni, C. , & Hansen, A. J. (2017). Algorithm for post‐clustering curation of DNA amplicon data yields reliable biodiversity estimates. Nature Communications, 8, 1188. 10.1038/s41467-017-01312-x PMC5662604 29084957

[men14023-bib-0026] Geiger, M. F. , Moriniere, J. , Hausmann, A. , Haszprunar, G. , Wägele, W. , Hebert, P. D. N. , & Rulik, B. (2016). Testing the global malaise trap program—How well does the current barcode reference library identify flying insects in Germany? Biodiversity Data Journal, 4, e10671. 10.3897/BDJ.4.e10671 PMC5136679 27932930

[men14023-bib-0027] Haase, P. , Frenzel, M. , Klotz, S. , Musche, M. , & Stoll, S. (2016). The long‐term ecological research (LTER) network: Relevance, current status, future perspective and examples from marine, freshwater and terrestrial long‐term observation. Ecological Indicators, 65, 1–3. 10.1016/j.ecolind.2016.01.040

[men14023-bib-0028] Habel, J. C. , Ulrich, W. , Segerer, A. H. , Greifenstein, T. , Knubben, J. , Morinière, J. , Bozicevic, V. , Günter, A. , & Hausmann, A. (2023). Insect diversity in heterogeneous agro‐environments of Central Europe. Biodiversity and Conservation, 32, 4665–4678. 10.1007/s10531-023-02717-5

[men14023-bib-0029] Hajibabaei, M. , Porter, T. M. , Wright, M. , & Rudar, J. (2019). COI metabarcoding primer choice affects richness and recovery of indicator taxa in freshwater systems. PLoS One, 14, e0220953. 10.1371/journal.pone.0220953 31513585 PMC6742397

[men14023-bib-0030] Hallmann, C. A. , Sorg, M. , Jongejans, E. , Siepel, H. , Hofland, N. , Schwan, H. , Stenmans, W. , Müller, A. , Sumser, H. , Hörren, T. , Goulson, D. , & de Kroon, H. (2017). More than 75 percent decline over 27 years in total flying insect biomass in protected areas. PLoS One, 12, e0185809. 10.1371/journal.pone.0185809 29045418 PMC5646769

[men14023-bib-0031] Hardulak, L. A. , Morinière, J. , Hausmann, A. , Hendrich, L. , Schmidt, S. , Doczkal, D. , Müller, J. , Hebert, P. D. N. , & Haszprunar, G. (2020). DNA metabarcoding for biodiversity monitoring in a national park: Screening for invasive and pest species. Molecular Ecology Resources, 20, 1542–1557. 10.1111/1755-0998.13212 32559020

[men14023-bib-0079] Hartop, E. , Srivathsanm, A. , Ronquistm, F. , … Meier, R. (2022) Towards large‐scale integrative taxonomy (LIT): Resolving the dataconundrum for dark taxa. Systematic Biology, 71, 1404–1422. 10.1093/sysbio/syac033 35556139 PMC9558837

[men14023-bib-0032] Hartop, E. (2021). A multi‐faceted approach to a “dark taxon”: The hyperdiverse and poorly known scuttle flies (Diptera: Phoridae) . http://urn.kb.se/resolve?urn=urn:nbn:se:su:diva‐192276

[men14023-bib-0033] Hausmann, A. , Segerer, A. H. , Greifenstein, T. , Knubben, J. , Morinière, J. , Bozicevic, V. , Doczkal, D. , Günter, A. , Ulrich, W. , & Habel, J. C. (2020). Toward a standardized quantitative and qualitative insect monitoring scheme. Ecology and Evolution, 10, 4009–4020. 10.1002/ece3.6166 32489627 PMC7244892

[men14023-bib-0034] Hebert, P. D. N. , Braukmann, T. W. A. , Prosser, S. W. J. , Ratnasingham, S. , deWaard, J. R. , Ivanova, N. V. , Janzen, D. H. , Hallwachs, W. , Naik, S. , Sones, J. E. , & Zakharov, E. V. (2018). A sequel to sanger: Amplicon sequencing that scales. BMC Genomics, 19, 219. 10.1186/s12864-018-4611-3 29580219 PMC5870082

[men14023-bib-0035] Hleap, J. S. , Littlefair, J. E. , Steinke, D. , Hebert, P. D. N. , & Cristescu, M. E. (2021). Assessment of current taxonomic assignment strategies for metabarcoding eukaryotes. Molecular Ecology Resources, 21, 2190–2203. 10.1111/1755-0998.13407 33905615

[men14023-bib-0036] Hobern, D. (2021). BIOSCAN: DNA barcoding to accelerate taxonomy and biogeography for conservation and sustainability. Genome, 64, 161–164. 10.1139/gen-2020-0009 32268069

[men14023-bib-0037] Hoppeler, F. , Tachamo Shah, R. D. , Shah, D. N. , Jähnig, S. C. , Tonkin, J. D. , Sharma, S. , & Pauls, S. U. (2016). Environmental and spatial characterisation of an unknown fauna using DNA sequencing—An example with Himalayan Hydropsychidae (Insecta: Trichoptera). Freshwater Biology, 61, 1905–1920. 10.1111/fwb.12824

[men14023-bib-0039] Huang, J. , Miao, X. , Wang, Q. , Menzel, F. , Tang, P. , Yang, D. , Wu, H. , & Vogler, A. P. (2022). Metabarcoding reveals massive species diversity of Diptera in a subtropical ecosystem. Ecology and Evolution, 12, e8535. 10.1002/ece3.8535 35127039 PMC8796913

[men14023-bib-0038] Høye, T. T. , Ärje, J. , Bjerge, K. , Hansen, O. L. P. , Iosifidis, A. , Leese, F. , Mann, H. M. R. , Meissner, K. , Melvad, C. , & Raitoharju, J. (2021). Deep learning and computer vision will transform entomology. Proceedings of the National Academy of Sciences of the United States of America, 118, e2002545117. 10.1073/pnas.2002545117 33431561 PMC7812775

[men14023-bib-0040] IPBES . (2019). Summary for policymakers of the global assessment report on biodiversity and ecosystem services. *Zenodo*, 10.5281/zenodo.3553579

[men14023-bib-0041] Iwaszkiewicz‐Eggebrecht, E. , Granqvist, E. , Buczek, M. , Prus, M. , Kudlicka, J. , Roslin, T. , Tack, A. J. M. , Andersson, A. F. , Miraldo, A. , Ronquist, F. , & Łukasik, P. (2023). Optimizing insect metabarcoding using replicated mock communities. Methods in Ecology and Evolution, 14, 1130–1146. 10.1111/2041-210X.14073 37876735 PMC10593422

[men14023-bib-0042] Jeliazkov, A. , Gavish, Y. , Marsh, C. J. , Geschke, J. , Brummitt, N. , Rocchini, D. , Haase, P. , Kunin, W. E. , & Henle, K. (2022). Sampling and modelling rare species: Conceptual guidelines for the neglected majority. Global Change Biology, 28, 3754–3777. 10.1111/gcb.16114 35098624

[men14023-bib-0043] Ji, Y. , Ashton, L. , Pedley, S. M. , Edwards, D. P. , Tang, Y. , Nakamura, A. , Kitching, R. , Dolman, P. M. , Woodcock, P. , Edwards, F. A. , Larsen, T. H. , Hsu, W. W. , Benedick, S. , Hamer, K. C. , Wilcove, D. S. , Bruce, C. , Wang, X. , Levi, T. , Lott, M. , … Yu, D. W. (2013). Reliable, verifiable and efficient monitoring of biodiversity via metabarcoding. Ecology Letters, 16, 1245–1257. 10.1111/ele.12162 23910579

[men14023-bib-0044] Karlsson, D. , Hartop, E. , Forshage, M. , Jaschhof, M. , & Ronquist, F. (2020). The Swedish malaise trap project: A 15 year retrospective on a countrywide insect inventory. Biodiversity Data Journal, 8, e47255. 10.3897/BDJ.8.e47255 32015667 PMC6987249

[men14023-bib-0045] Keck, F. , & Altermatt, F. (2023). Management of DNA reference libraries for barcoding and metabarcoding studies with the R package refdb. Molecular Ecology Resources, 23, 511–518. 10.1111/1755-0998.13723 36239541

[men14023-bib-0046] Klausnitzer, B. (2005). Die Insektenfauna Deutschlands (“Entomofauna Germanica”)‐ein Gesamtüberblick. Linzer Biologische Beiträge, 37, 87–97.

[men14023-bib-0047] Li, M. , Lei, T. , Wang, G. , Zhang, D. , Liu, H. , & Zhang, Z. (2023). Monitoring insect biodiversity and comparison of sampling strategies using metabarcoding: A case study in the Yanshan Mountains, China. Ecology and Evolution, 13, e10031. 10.1002/ece3.10031 37091562 PMC10121320

[men14023-bib-0048] Marquina, D. , Roslin, T. , Łukasik, P. , & Ronquist, F. (2022). Evaluation of non‐destructive DNA extraction protocols for insect metabarcoding: Gentler and shorter is better. Metabarcoding and Metagenomics, 6, e78871. 10.3897/mbmg.6.78871

[men14023-bib-0049] Martin, M. (2011). Cutadapt removes adapter sequences from high‐throughput sequencing reads. EMBnet.Journal, 17, 10–12. 10.14806/ej.17.1.200

[men14023-bib-0050] McGee, K. M. , Robinson, C. V. , & Hajibabaei, M. (2019). Gaps in DNA‐based biomonitoring across the globe. Frontiers in Ecology and Evolution, 7. 10.3389/fevo.2019.00337

[men14023-bib-0051] Meier, R. , Wong, W. , Srivathsan, A. , & Foo, M. (2016). $1 DNA barcodes for reconstructing complex phenomes and finding rare species in specimen‐rich samples. Cladistics, 32, 100–110. 10.1111/cla.12115 34732017

[men14023-bib-0052] Meiklejohn, K. A. , Damaso, N. , & Robertson, J. M. (2019). Assessment of BOLD and GenBank—Their accuracy and reliability for the identification of biological materials. PLoS One, 14, e0217084. 10.1371/journal.pone.0217084 31216285 PMC6584008

[men14023-bib-0053] Mirtl, M. , Borer, E. T. , Djukic, I. , Forsius, M. , Haubold, H. , Hugo, W. , Jourdan, J. , Lindenmayer, D. , McDowell, W. H. , Muraoka, H. , Orenstein, D. E. , Pauw, J. C. , Peterseil, J. , Shibata, H. , Wohner, C. , Yu, X. , & Haase, P. (2018). Genesis, goals and achievements of long‐term ecological research at the global scale: A critical review of ILTER and future directions. Science of the Total Environment, 626, 1439–1462. 10.1016/j.scitotenv.2017.12.001 29898550

[men14023-bib-0054] Montgomery, G. A. , Belitz, M. W. , Guralnick, R. P. , & Tingley, M. W. (2021). Standards and best practices for monitoring and benchmarking insects. Frontiers in Ecology and Evolution, 8. 10.3389/fevo.2020.579193

[men14023-bib-0055] Pawlowski, J. , Bruce, K. , Panksep, K. , Aguirre, F. I. , Amalfitano, S. , Apothéloz‐Perret‐Gentil, L. , Baussant, T. , Bouchez, A. , Carugati, L. , Cermakova, K. , Cordier, T. , Corinaldesi, C. , Costa, F. O. , Danovaro, R. , Dell'Anno, A. , Duarte, S. , Eisendle, U. , Ferrari, B. J. D. , Frontalini, F. , … Fazi, S. (2022). Environmental DNA metabarcoding for benthic monitoring: A review of sediment sampling and DNA extraction methods. Science of the Total Environment, 818, 151783. 10.1016/j.scitotenv.2021.151783 34801504

[men14023-bib-0056] Pereira, C. L. , Gilbert, M. T. P. , Araújo, M. B. , & Matias, M. G. (2021). Fine‐tuning biodiversity assessments: A framework to pair eDNA metabarcoding and morphological approaches. Methods in Ecology and Evolution, 12, 2397–2409. 10.1111/2041-210X.13718

[men14023-bib-0057] Ratnasingham, S. , & Hebert, P. D. N. (2007). bold: The barcode of life data system (http://www.barcodinglife.org). Molecular Ecology Notes, 7, 355–364. 10.1111/j.1471-8286.2007.01678.x 18784790 PMC1890991

[men14023-bib-0058] Ratnasingham, S. , & Hebert, P. D. N. (2013). A DNA‐based registry for all animal species: The barcode index number (BIN) system. PLoS One, 8, e66213. 10.1371/journal.pone.0066213 23861743 PMC3704603

[men14023-bib-0059] Resh, V. , & Jackson, J. (1993). Rapid assessment approaches to biomonitoring using benthic macroinvertebrates. In D. M. Rosenberg & V. H. Resh (Eds.), Freshwater biomonitoring and benthic macroinvertebrates (pp. 195–233). Chapman & Hall.

[men14023-bib-0060] Rognes, T. , Flouri, T. , Nichols, B. , Quince, C. , & Mahé, F. (2016). VSEARCH: A versatile open source tool for metagenomics. PeerJ, 4, e2584. 10.7717/peerj.2584 27781170 PMC5075697

[men14023-bib-0061] Ronquist, F. , Forshage, M. , Häggqvist, S. , Karlsson, D. , Hovmöller, R. , Bergsten, J. , Holston, K. , Britton, T. , Abenius, J. , Andersson, B. , Buhl, P. N. , Coulianos, C.‐C. , Fjellberg, A. , Gertsson, C.‐A. , Hellqvist, S. , Jaschhof, M. , Kjærandsen, J. , Klopfstein, S. , Kobro, S. , … Gärdenfors, U. (2020). Completing Linnaeus's inventory of the Swedish insect fauna: Only 5,000 species left? PLoS One, 15, e0228561. 10.1371/journal.pone.0228561 32130216 PMC7055846

[men14023-bib-0062] Sickel, W. , Zizka, V. , Scherges, A. , Bourlat, S. J. , & Dieker, P. (2023). Abundance estimation with DNA metabarcoding—Recent advancements for terrestrial arthropods. Metabarcoding and Metagenomics, 7, e112290. 10.3897/mbmg.7.112290

[men14023-bib-0063] Srivathsan, A. , Ang, Y. , Heraty, J. M. , Hwang, W. S. , Jusoh, W. F. A. , Kutty, S. N. , Puniamoorthy, J. , Yeo, D. , Roslin, T. , & Meier, R. (2023). Convergence of dominance and neglect in flying insect diversity. Nature Ecology & Evolution, 7, 1012–1021. 10.1038/s41559-023-02066-0 37202502 PMC10333119

[men14023-bib-0064] Steinke, D. , Braukmann, T. W. , Manerus, L. , Woodhouse, A. , & Elbrecht, V. (2021). Effects of Malaise trap spacing on species richness and composition of terrestrial arthropod bulk samples. Metabarcoding and Metagenomics, 5, e59201. 10.3897/mbmg.5.59201

[men14023-bib-0065] Stork, N. E. (2018). How many species of insects and other terrestrial arthropods are there on Earth? Annual Review of Entomology, 63, 31–45. 10.1146/annurev-ento-020117-043348 28938083

[men14023-bib-0066] Sturmbauer, C. , Opadiya, G. B. , Niederstätter, H. , Riedmann, A. , & Dallinger, R. (1999). Mitochondrial DNA reveals cryptic oligochaete species differing in cadmium resistance. Molecular Biology and Evolution, 16, 967–974. 10.1093/oxfordjournals.molbev.a026186 10406113

[men14023-bib-0067] Telenius, A. (2011). Biodiversity information goes public: GBIF at your service. Nordic Journal of Botany, 29, 378–381. 10.1111/j.1756-1051.2011.01167.x

[men14023-bib-0068] Uhler, J. , Redlich, S. , Zhang, J. , Hothorn, T. , Tobisch, C. , Ewald, J. , Thorn, S. , Seibold, S. , Mitesser, O. , Morinière, J. , Bozicevic, V. , Benjamin, C. S. , Englmeier, J. , Fricke, U. , Ganuza, C. , Haensel, M. , Riebl, R. , Rojas‐Botero, S. , Rummler, T. , … Müller, J. (2021). Relationship of insect biomass and richness with land use along a climate gradient. Nature Communications, 12, 5946. 10.1038/s41467-021-26181-3 PMC8511018 34642336

[men14023-bib-0069] Vamos, E. , Elbrecht, V. , & Leese, F. (2017). Short COI markers for freshwater macroinvertebrate metabarcoding. Metabarcoding and Metagenomics, 1, e14625. 10.3897/mbmg.1.14625

[men14023-bib-0070] van Klink, R. , August, T. , Bas, Y. , Bodesheim, P. , Bonn, A. , Fossøy, F. , Høye, T. T. , Jongejans, E. , Menz, M. H. M. , Miraldo, A. , Roslin, T. , Roy, H. E. , Ruczyński, I. , Schigel, D. , Schäffler, L. , Sheard, J. K. , Svenningsen, C. , Tschan, G. F. , Wäldchen, J. , … Bowler, D. E. (2022). Emerging technologies revolutionise insect ecology and monitoring. Trends in Ecology & Evolution, 37, 872–885. 10.1016/j.tree.2022.06.001 35811172

[men14023-bib-0071] Wagner, D. L. (2020). Insect declines in the Anthropocene. Annual Review of Entomology, 65, 457–480. 10.1146/annurev-ento-011019-025151 31610138

[men14023-bib-0072] Welti, E. A. R. , Zajicek, P. , Frenzel, M. , Ayasse, M. , Bornholdt, T. , Buse, J. , Classen, A. , Dziock, F. , Engelmann, R. A. , Englmeier, J. , Fellendorf, M. , Förschler, M. I. , Fricke, U. , Ganuza, C. , Hippke, M. , Hoenselaar, G. , Kaus‐Thiel, A. , Kerner, J. , Kilian, D. , … Haase, P. (2022). Temperature drives variation in flying insect biomass across a German malaise trap network. Insect Conservation and Diversity, 15, 168–180. 10.1111/icad.12555

[men14023-bib-0073] Wetzel, F. T. , Saarenmaa, H. , Regan, E. , Martin, C. S. , Mergen, P. , Smirnova, L. , Tuama, É. Ó. , García Camacho, F. A. , Hoffmann, A. , Vohland, K. , & Häuser, C. L. (2015). The roles and contributions of biodiversity observation networks (BONs) in better tracking progress to 2020 biodiversity targets: A European case study. Biodiversity, 16, 137–149. 10.1080/14888386.2015.1075902

[men14023-bib-0074] Wilkinson, M. D. , Dumontier, M. , IjJ, A. , Appleton, G. , Axton, M. , Baak, A. , Blomberg, N. , Boiten, J.‐W. , da Silva Santos, L. B. , Bourne, P. E. , Bouwman, J. , Brookes, A. J. , Clark, T. , Crosas, M. , Dillo, I. , Dumon, O. , Edmunds, S. , Evelo, C. T. , Finkers, R. , … Mons, B. (2016). The FAIR guiding principles for scientific data management and stewardship. Scientific Data, 3, 160018. 10.1038/sdata.2016.18 26978244 PMC4792175

[men14023-bib-0075] Wührl, L. , Pylatiuk, C. , Giersch, M. , Lapp, F. , von Rintelen, T. , Balke, M. , Schmidt, S. , Cerretti, P. , & Meier, R. (2022). DiversityScanner: Robotic handling of small invertebrates with machine learning methods. Molecular Ecology Resources, 22, 1626–1638. 10.1111/1755-0998.13567 34863029

[men14023-bib-0076] Zizka, V. M. A. , Elbrecht, V. , Macher, J.‐N. , & Leese, F. (2019). Assessing the influence of sample tagging and library preparation on DNA metabarcoding. Molecular Ecology Resources, 19, 893–899. 10.1111/1755-0998.13018 30963710

[men14023-bib-0077] Zizka, V. M. A. , Geiger, M. F. , Hörren, T. , Kirse, A. , Noll, N. W. , Schäffler, L. , Scherges, A. M. , & Sorg, M. (2022). Repeated subsamples during DNA extraction reveal increased diversity estimates in DNA metabarcoding of Malaise traps. Ecology and Evolution, 12, e9502. 10.1002/ece3.9502 36447594 PMC9702565

[men14023-bib-0078] Zizka, V. M. A. , Koschorreck, J. , Khan, C. C. , & Astrin, J. J. (2022). Long‐term archival of environmental samples empowers biodiversity monitoring and ecological research. Environmental Sciences Europe, 34, 40. 10.1186/s12302-022-00618-y

