# Supplementary figures and images for: Upscaling biodiversity monitoring: Metabarcoding estimates 31,846 insect species from Malaise traps across Germany
Source: Mol Ecol Resour. 2024 Oct 4;25(1):e14023. doi: 10.1111/1755-0998.14023 (PMC11646302; doi:10.1111/1755-0998.14023)

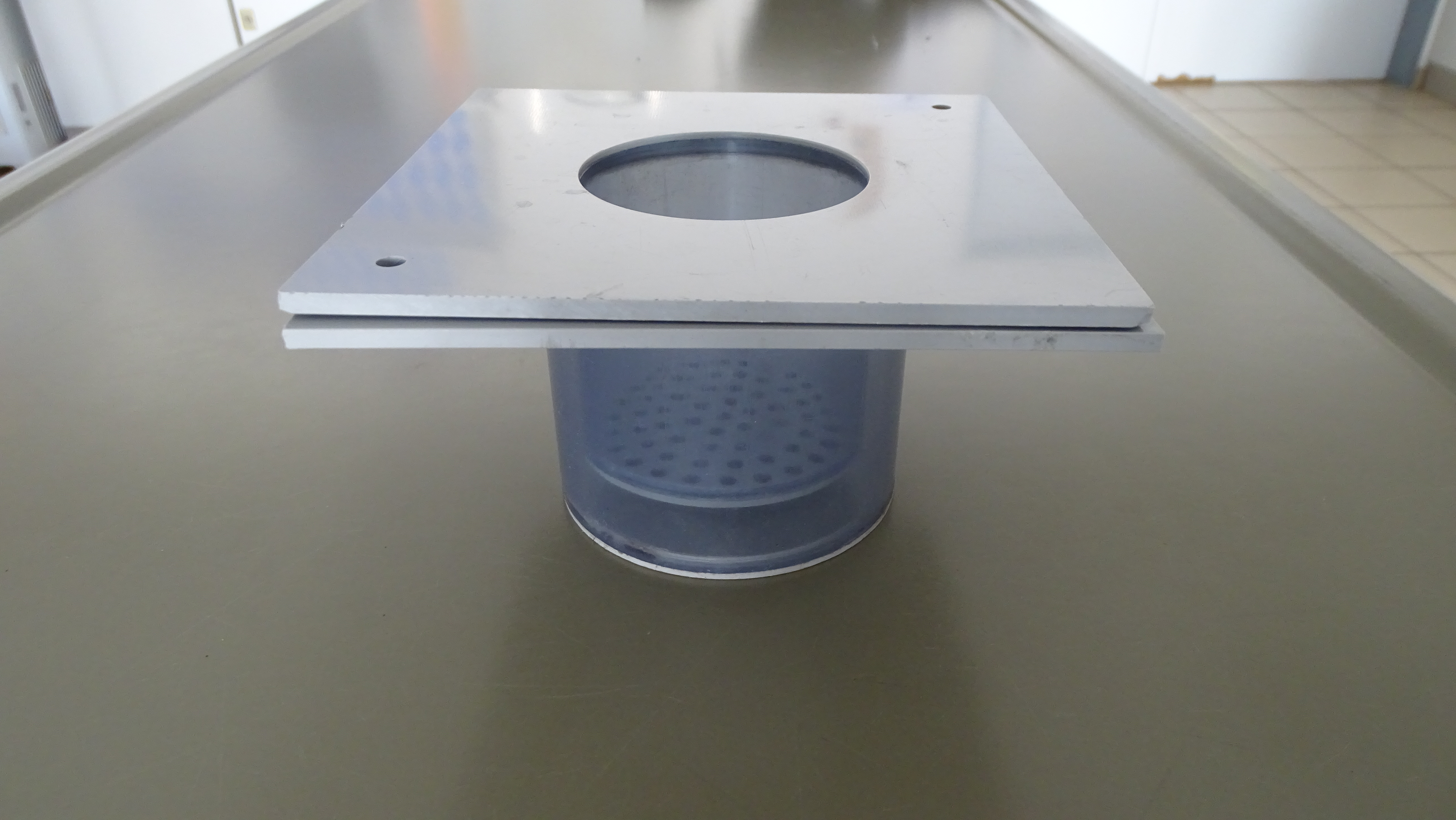

Supplement: Supplementary file 1 — Appendix S1. [file MEN-25-e14023-s001.zip › LTER Supplement/Figure S1.JPG]

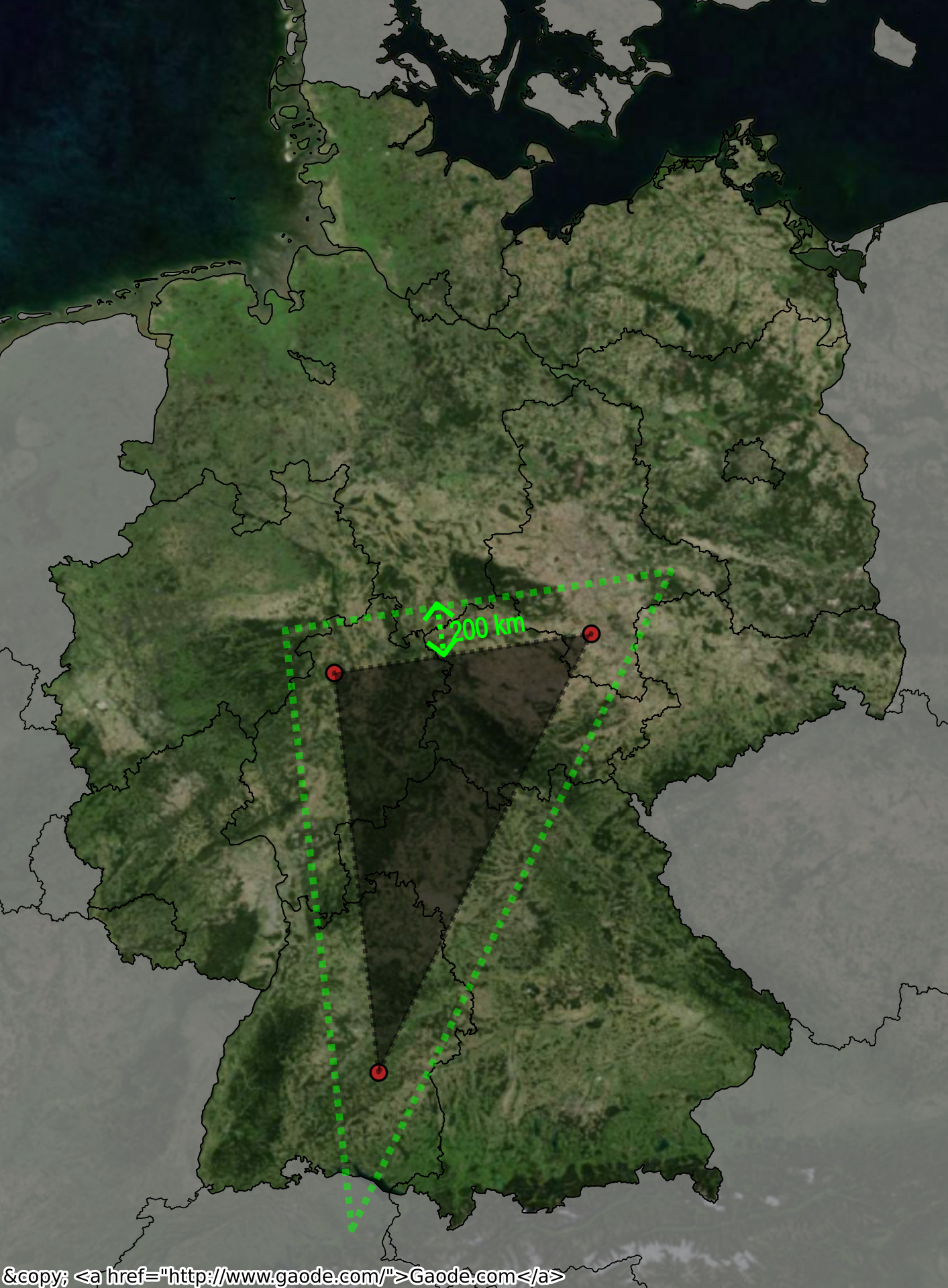

Supplement: Supplementary file 1 — Appendix S1. [file MEN-25-e14023-s001.zip › LTER Supplement/Figure S2.png]
